# Short and longer-term impacts of health insurance on catastrophic health expenditures in Kwara State, Nigeria

**DOI:** 10.1186/s12913-022-08917-z

**Published:** 2022-12-20

**Authors:** Adeyemi Okunogbe, Joel Hähnle, Bosede F. Rotimi, Tanimola M. Akande, Wendy Janssens

**Affiliations:** 1 Global Health Division, RTI International, Washington, DC. USA; 2 Amsterdam Institute for Global Health and Development (AIGHD), De Boelelaan 1105, 1081 HV Amsterdam, The Netherlands; 3 Department of Epidemiology and Community Health, University of Ilorin, Ilorin, Nigeria; 4 School of Business and Economics, Vrije Universiteit Amsterdam, Amsterdam, The Netherlands

**Keywords:** Catastrophic health expenditures, Health insurance, Out-of-pocket payments, Universal health coverage, Nigeria, Sub-Saharan Africa

## Abstract

**Background:**

Out- of-pocket health expenditures (OOPs) constitute a significant proportion of total health expenditures in many low- and middle-income countries (LMICs), leading to an increased likelihood of exposure to financial catastrophe in the event of illness. Health insurance has the potential to reduce catastrophic health expenditures (CHE), but rigorous evidence of its sustained impact is limited, especially in LMICs. This study examined the short- and longer-term effects of a health insurance program in Kwara State, Nigeria on CHE.

**Methods:**

The analysis is based on a panel dataset consisting of 3 waves of household surveys in program and comparison areas. The balanced data consists of 1,039 households and 3,450 individuals. We employed a difference-in-differences (DiD) regression approach to estimate intention-to-treat effects, and then computed average treatment effects on the treated by combining DiD with propensity score weighting and an instrumental variables analysis. CHE was measured as OOPs exceeding 10% of household consumption and 40% of capacity-to-pay (CTP).

**Results:**

Using 10% of consumption as a CHE measure, we found that living in the program area was associated with a 4.3 percentage point (pp) decrease in CHE occurrence (*p* < 0.05), while the effect on insured households was 5.7 pp (*p* < 0.05). The longer-term impact four years after program introduction was not significant. Heterogeneity analyses show a reduction in CHE of 7.2 pp (*p* < 0.01) in the short-term for the poorest tercile. No significant effects were found for the middle and richest terciles, nor in the longer-term. Households with a chronically ill member experienced a reduction in CHE of 9.4 pp (*p* < 0.01) in the short-term, but not in the longer-term. Most estimates based on the 40% of CTP measure were not statistically significant.

**Conclusion:**

These findings highlight the critical role of health insurance in reducing the likelihood of catastrophic health expenditures, especially for vulnerable populations such as the poor and the chronically ill, and by extension in achieving universal health coverage. They also show that the beneficial impacts of health insurance may attenuate over time, as households potentially adjust their health-seeking behavior to the new scheme.

**Supplementary information:**

The online version contains supplementary material available at 10.1186/s12913-022-08917-z.

## Background

Out-of-pocket expenditures (OOPs) constitute a significant proportion of health expenditures in many low- and middle-income countries (LMICs) leading to an increased likelihood of exposure to financial catastrophe in the event of illness. Health spending is said to be catastrophic when OOPs reach a certain threshold of household expenditures. Evidence suggests that almost one billion individuals worldwide suffer financial catastrophe annually, while 70 million are pushed below the extreme poverty line due to catastrophic health spending [1].

The World Health Organization (WHO) and the World Bank estimated the global incidence of catastrophic health expenditures (CHE, defined as spending more than 10% of household consumption on health) to be 13.2% in 2017—equivalent to about 996 million people, rising from 579 million in 2000 and 785 million in 2010 [1]. They also find the highest concentration of catastrophic health spending in the world’s poorest regions – Asia and Africa, at 16.6% and 10.0%, respectively. These regions were reported to have had an incidence of impoverishment at the $1.90 per day poverty line (i.e. households being pushed below the extreme poverty line) of 1.1% and 1.4%, respectively, accounting for 98.5% of the world’s population impoverished by OOPs in 2017; while 3.1% and 23.4% of their populations, respectively, were pushed *further* below the poverty line [1]. Hence, the likelihood of impoverishment due to OOPs appears high in countries with high poverty rates, feeding a vicious cycle of worsened poverty and ill-health [2].

There is global consensus that achieving universal health coverage (UHC) – one of the goals of the United Nations Sustainable Development Agenda (SDG 3.8) – is critical to reducing financial risk for households. UHC is defined as ensuring that everyone is able to access needed health care services of adequate quality without suffering undue financial hardship as a result [3]. Formal health insurance mechanisms play a significant role in ensuring financial risk protection [4–10]. Without access to health insurance, households may need to resort to alternative risk-coping strategies in order to pay for their medical bills, such as selling productive assets (e.g. livestock), taking children out of school, borrowing from informal money lenders or depleting savings – strategies that are often harmful to their future welfare [6, 11–14]. In this context, reductions in CHE are often used as a proxy for financial protection, as they can be calculated with widely available data, thereby allowing comparisons across populations and over time in terms of progress towards SDG 3.8.

We recognize that for some households, it is the *absolute* level of spending that matters, as they are so poor that even small levels of OOPs may push them further into poverty, regardless of whether these expenditures reach the threshold of 10% of household income [1]. Moreover, sick individuals might also decide to forego care all together, thereby leading to dismal health outcomes. Measurements of impoverishment and foregone care due to inability to pay are beyond the scope of these analyses.

Access to health insurance is particularly low in Sub-Saharan Africa (SSA). This paper seeks to examine how the provision of voluntary community-based health insurance impacts on CHE in Nigeria—a lower middle-income country with particularly low health insurance coverage. Whereas an extensive literature documents the impact of health insurance on OOPs in LMICs (see [15, 16] for overviews), evidence on the impact of health insurance on CHE in LMICs is limited with some significant gaps. First, most of the previous research, especially for the Sub-Saharan African region, is based on cross-sectional studies. Evidence from more rigorous methods such as longitudinal panel studies is mostly drawn from other regions, notably Latin-America and Asia. Second, the existing evidence for SSA is mixed and inconclusive, as described below. Third, most longitudinal studies either focus on the impact of health insurance in the short-term (immediately after the introduction of the scheme) or in the longer-term, but they do not examine both with repeated assessments, and hence do not provide insights into sustainability of impacts over time.

Cross-sectional studies of the impact of insurance on CHE in SSA have been conducted amongst others in Kenya, Ghana, Ethiopia, Nigeria and Zambia. A simulation study of the *National Hospital Insurance Fund* (NHIF) in Kenya estimated that health insurance would decrease the risk of CHE more drastically for beneficiaries in lower wealth quintiles than for higher quintiles in absolute terms [17]. However, Xu et al. (2006) did not find a significant impact of the NHIF [18]. After the implementation of the *National Health Insurance Scheme* in Ghana, Nguyen et al. (2011) and Navarrete et al. (2019) found a significant reduction in the probability of beneficiaries experiencing CHE at the 20% and 10% threshold of capacity to pay, respectively [19, 20]. Saksena et al. (2010) and Mekonen et. al (2018) evaluated the community-based health insurance schemes (*Mutuelles)* in Rwanda and Ethiopia, respectively, and also found significant reductions in CHE among the insured [21, 22]. On the other hand, Ilesanmi et al. (2014) found no significant reduction in the likelihood of CHE among insured households in Southwestern Nigeria, while Ekman (2007) in an evaluation of a pre-payment scheme in Zambia found evidence actually suggesting an increase in the risk of CHE [23, 24]. This mixed and inconclusive evidence is in line with the findings from cross-sectional studies in other regions such as Asia [8, 25–27] and Latin America [28–30].

Evidence from more rigorous, longitudinal studies in SSA is scarce. Aryeetey et al. (2016) evaluated the short-term impact of the *National Health Insurance Scheme* in Ghana on CHE with household survey data from a baseline in 2009 to 2011 and found that insurance coverage decreased the likelihood of CHE by 3% [31]. A review of (quasi-) experimental studies from other regions of the world yields mixed insights. Bernal et al. (2017) find significant evidence of CHE reductions due to social health insurance in Peru [32]. King et al. (2009), Galárraga et al. (2010) and Grogger et al. (2014) evaluated the short-term impact of the landmark health insurance program *Seguro Popular* in Mexico, finding evidence that the insurance program reduced CHE [33–35]. However, a study of the longer-term effect of *Seguro Popular* using repeated cross-sections over a time period of eight years found no significant effects on the incidence of CHE [36]. In Asia, Limwattananon et al. (2015) find a significant reduction in CHE after a major reform towards universal health insurance coverage [37]. Axelson et al. (2009) observed a reduced risk of CHE after the introduction of the *Health Care Fund for the Poor* in Vietnam [38]; and Xie et al. (2018) report a reduction in CHE from their evaluation of the Chinese New Cooperative Medical Scheme [39]. While Barnes et al. (2017) found a decrease in CHE from the *Vajpayee Arogyashree Scheme* in India, other studies of Indian health insurance schemes found no significant effects [40–42]. Notably, both Wagstaff and Lindelow (2008) and Sparrow et al. (2013) found that health insurance enrollment actually increased the risk of CHE in China and Indonesia, respectively [43, 44]. The authors hypothesize that these counterintuitive findings might have been due to an increased demand for healthcare as facilitated by health insurance, leading to additional associated costs not covered by the insurance scheme, such as transportation costs or medicines.

This paper examines the impact of health insurance on catastrophic health expenditure (CHE) in Kwara State, Nigeria and investigates heterogeneity of impacts by baseline wealth and health. The study contributes to the existing literature in two main ways. First, we study the impact on CHE over both a two- and a four-year period, whereas most previous studies focus on estimating impact at one point in time. We are therefore able to examine not only the immediate impacts of introducing health insurance on CHE, but also to what extent short-term impacts realized soon after program roll-out are sustained, reinforced or fade further out after the program’s introduction. Thus, we measure and compare impact estimates repeatedly. Second, unlike most existing studies in SSA, we use rich longitudinal quasi-experimental data collected from a panel of households in three waves. This enables us to adjust for unobserved time-invariant characteristics that may be correlated with health insurance uptake and may bias cross-sectional estimates. The findings of this study hence contribute to the evidence-base on the impacts of health insurance on CHE, one of the most widely used proxies for protection against health-related financial risk.

## Context and Intervention

The geographic context of the study is Kwara State in central Nigeria. Nigeria is the most populous country in Africa with a population of over 200 million; and has amongst the worst health indicators, having the 4^th^ highest maternal mortality and 12^th^ highest infant mortality rate in the world [45]. Over 70% of Nigerian households finance health expenditures out-of-pocket [46]. Only 3% of Nigerians are enrolled in formal health insurance, mainly through the National Health Insurance Scheme (NHIS), a social health insurance scheme established by the federal government and launched in 2005 [47–50]. Kwara State has a population of over 3 million with a predominantly agrarian economy and a poverty head count of 72% at the start of the study period in 2009 [51].

In 2007, the Nigerian health maintenance organization Hygeia Ltd. with support from the Dutch Health Insurance Fund and PharmAccess Foundation introduced a community-based health insurance program in Kwara state – the Kwara State Health Insurance Program (KSHIP). The program subsidized the cost of annual premiums for enrollees and upgraded selected health facilities in the intervention areas, hence targeting both the demand- and supply-side challenges of health care access. Enrolment in the insurance program was at the individual level and could occur on a monthly basis. Coverage lasted for a full year, after which it needed to be renewed. Enrollment and renewal were voluntary. Subsidies were high during the study period, with annual co-payments for enrollees between US$ 3 and US$ 5 per individual. Nevertheless, for the large and poor households in Kwara State, enrolling an entire family may still have been a significant lump-sum expenditure.

Prior evaluations of KSHIP have found that it had a significant impact on improving health care utilization and health outcomes, as well as reducing out-of-pocket spending in the short-term but not in the longer term [52–54]. In 2018, the state government mandated health insurance by law and committed to replicating the KSHIP model across the state [55]. In 2020, the new state-funded program was rolled-out.

## Methods

### Study design

Data for this study is from a survey of a randomly selected representative sample of households in intervention and control areas. The survey has been previously published as part of an impact evaluation [53]. The intervention area comprised two districts (Afon and Abota Oja) where the insurance program was introduced after the baseline survey. A third district (Ajasse Ipo) was included as the control area and was selected to be as similar as possible to the intervention area on key demographic, socio-economic and health indicators.

Using a two-stage stratified random sampling design at baseline in 2009, 100 out of 300 enumerations areas (EAs) were randomly selected (first stage). Only EAs within a 15-km radius of the main health facility in each district were included in the sampling frame. Forty EAs were sampled in the control district, and 30 EAs in each of the two program districts. Next, an average of 15 households per EA were randomly selected from a census of households in the selected EAs, with the exact numbers proportional to EA size (second stage). Other details about the survey design have been extensively described elsewhere [52, 56, 57]. A total of 1,450 households were interviewed at baseline, of which 884 were residing in the treatment area and 566 were residing in the control area.

Follow-up surveys of the households interviewed at baseline were conducted in 2011 and 2013. Tracking rates were reasonable for a long-term follow-up at 72% of the original sample in 2013. Our analysis focuses on the balanced panel of 1,039 households (672 in the treatment and 367 in the control area), made up of 2,210 individuals in the intervention area and 1,240 individuals in the control area.

The surveys collected information on characteristics such as demographics, health insurance status, health care status and utilization, health care expenditures, household consumption and income.

### Key outcome variable

Our key outcome variable is the occurrence of catastrophic health expenditures (CHE). This is a binary variable coded as 1 for occurrence of CHE in the year prior to the survey, and 0 otherwise. Out-of-pocket health expenditures are said to be catastrophic when they exceed a given percentage of household income or consumption [58]. Thresholds that have been used in past studies include 10% and 25% of total annual household consumption, with 10% being more commonly used [1, 59]. The choice of the denominator depends on availability of data and geographic context. For example, income data may not be very reliable in LMICs; hence, total expenditures are typically used as the denominator. Another approach involves using the household’s capacity to pay (CTP) as the denominator—defined as the effective income left after basic subsistence needs have been accounted for [60]; with a standard threshold of 40%. Basic subsistence needs are calculated as the average food expenditures of households whose food share is in the 45^th^ to 55^th^ percentile range. When more than 40% of the calculated capacity to pay is spent on health care in a given period by a household, then such health expenditures are deemed to be catastrophic [60, 61].

In line with the most recent international reports on financial protection [1], we operationalized CHE as OOPs exceeding 10% of household consumption. We used the total annual household expenditures as the denominator of this variable. This so-called ‘budget share’ approach is our preferred measure. As a robustness check, we also calculated CHE as OOPs exceeding 40% of household CTP. Finally, we test the sensitivity of our main results to varying threshold levels for both measures.

### Independent variables

The first key independent variable is the treatment indicator – measuring whether a household is residing in the treatment area, with access to the insurance program, or in the control area. The treatment indicator encompasses both insured and uninsured households, as not everyone in the treatment area enrolled in the program. The insured benefit both from improved financial access and improved quality of care at upgraded program facilities, the uninsured benefit only from the improved quality of care. The second key independent variable is health insurance status. This is a choice variable, since enrolment in health insurance was voluntary. Insurance status is recorded in the data at the individual level. More specifically, an individual is defined to be insured when he or she was insured at any point in time in the 12 months prior to each survey. Enrolment in insurance schemes other than KSHIP was negligible at less than 1% over the entire study period. The data on health expenditures are recorded on an annual basis and at the household level, therefore we are not able to link health expenditures to individual monthly insurance status. The household insurance indicator is set equal to 1 if at least one household member was enrolled in KSHIP health insurance in the year prior to the survey, and 0 otherwise. It should be noted that households did not necessarily enroll all their household members. This will yield a lower bound on the insurance impact estimates. Other relevant independent variables included as covariates include demographic, socio-economic and health-related factors as shown in Table 1.

**Table 1:** Baseline Household Characteristics of Balanced panel: 2009 (Treatment and Control Areas), 2011 & 2013 (Insured and Uninsured in Treatment Area)

	**2009** **Total** **(1)**	**2009** **Control** **(2)**	**2009** **Treatment** **(3)**	**Diff. of means** **(4)**	**2011 Treatment and Not Insured** **(5)**	**2011 Treatment and Insured** **(6)**	**Diff. of means** **(7)**	**2013 Treatment and Not Insured** **(8)**	**2013 Treatment and Insured** **(9)**	**Diff. of means** **(10)**
*Demographic characteristics*										
Age of HH head	52.11	49.46	53.56	-4.096***	55.96	54.98	-0.984	57.15	57.78	0.627
Household head is Female	0.221	0.278	0.190	0.088***	0.231	0.211	-0.020	0.291	0.212	-0.079**
Dummy: household head is married	0.796	0.775	0.807	-0.031	0.769	0.825	0.056*	0.696	0.789	0.093**
Household size	4.437	4.431	4.440	-0.010	4.504	4.725	0.221	3.909	4.657	0.748***
*Socio-economic characteristics*										
HH head: At least primary school education	0.559	0.676	0.496	0.180***	0.447	0.554	0.107**	0.470	0.520	0.051
Dummy: household head has worked in past year	0.930	0.932	0.929	0.003	0.933	0.928	-0.006	0.929	0.883	-0.047**
Annual non-medical consumption of household (in Naira)	560,917	602,190	538,377	63,813	453,931	573,795	119,864***	435,809	520,082	84,273**
Wealth Index (1–3)	1.999	2.232	1.872	0.360***	1.706	2.072	0.366***	1.747	1.991	0.244***
Wealth Index = Poor	0.334	0.221	0.396	0.175***	0.488	0.285	-0.202***	0.448	0.346	-0.102**
Wealth Index = Middle	0.333	0.327	0.336	0.009	0.319	0.357	0.039	0.357	0.317	-0.040
Wealth Index = Rich	0.333	0.452	0.268	0.184***	0.193	0.357	0.164***	0.195	0.337	0.142***
Good quality toilet	0.081	0.202	0.015	0.187***	0.005	0.0393	0.034**	0.009	0.0349	0.026*
Good quality drinking water	0.808	0.894	0.760	0.133***	0.768	0.800	0.032	0.777	0.797	0.019
Urban: Household lives in a town vs village	0.519	0.572	0.490	0.083	0.357	0.649	0.292***	0.424	0.552	0.129**
*Health-related characteristics*										
Distance (km) to nearest HCHP clinic	3.158	3.655	2.887	0.768	3.616	2.386	-1.230***	3.536	2.794	-0.742**
Distance (km) to nearest clinic (all 79 clinics)	1.191	1.185	1.194	-0.001	1.409	1.133	-0.277	1.324	1.328	0.004
Dummy: All HH members can do daily activities without difficulty	0.311	0.365	0.281	0.084**	0.259	0.216	-0.042	0.329	0.215	-0.114***
Dummy: at least 1 HH member has a chronic disease	0.218	0.234	0.210	0.025	0.332	0.390	0.058	0.326	0.462	0.136***
Dummy: at least 1 HH member had an acute illness/injury in past 12 months	0.400	0.447	0.375	0.072	0.499	0.636	0.137**	0.628	0.770	0.142***
HH annual health expenditures (excl. premium)	4,809	4,748	4,843	-95.48	3,752	3,809	56.947	6,125	7,125	1,000
Dummy at least one person in household is insured	0.001	0.008	0.010	-0.002	0	1	1.000***	0	1	1.000
Number of Observations	1,039	367	672	1,039	367	305	672	328	344	672

^*^ p < 0.10; ** p < 0.05; *** p < *0.01.* Robust standard errors clustered at EA level. Based on the balanced panel of 1,039 households and 3,450 individuals

### Empirical Strategy

Our estimation of the effect of health insurance on the probability of CHE draws on two approaches: First, we estimate the impact of exposure to the intervention, i.e. residing in the treatment area, and thereby having access to subsidized health insurance and upgraded health facilities, compared to residing in the control area without such access. This is an *intention-to-treat* (ITT) analysis that provides an estimate of the differential effect of the intervention on those in the treatment area, regardless of their actual insurance status.

We employ a difference-in-differences (DiD) strategy to analyze the short-term impact between the baseline in 2009 and the follow-up survey in 2011 to capture changes soon after insurance roll-out, and the longer-term impact between the baseline in 2009 and the follow-up survey in 2013 to evaluate whether program impacts were sustained, reinforced or attenuated four years after its introduction.

The difference-in-differences approach is specified using a fixed effect linear regression model as shown below:

1 $${Y}_{it}=\beta .{D}_{it} {{+ X}_{it}}^{^{\prime}}\theta +{{\gamma }_{i}+{\varphi }_{t}+\varepsilon }_{it}$$

where ${Y}_{it}$ denotes the binary outcome variable (CHE measured either as 10% of household consumption or as 40% of CTP) for household *i* in year *t*; ${D}_{it}$ is an indicator variable that takes on the value 1 if an household resides in the treatment area and 0 otherwise interacted with the time fixed effect; ${X}_{it}$ is a vector of relevant time-variant covariates; ${\gamma }_{i}$ captures household fixed effects; ${\varphi }_{t}$ captures time fixed effects; and ${\varepsilon }_{it}$ is the independently distributed error term. Standard errors are robust and clustered at the enumeration area (EA) level. The included covariates are gender and marital status of the household head, and household size (Table 1). The coefficient $\beta$ is the DiD estimate of the impact of living in the treatment area on CHE occurrence (comparing baseline with either 2011 or 2013). This DiD approach assumes parallel trends in outcomes between the treatment and control group in the absence of the intervention.

The ITT approach estimates the effect of residing in the treatment area on CHE irrespective of health insurance status. This is an important outcome from a policy perspective because it shows the aggregate effect of offering voluntary subsidized health insurance combined with quality upgrades to a target population, taking into account that not every household will choose to enroll. However, it does not show the impact of health insurance on the insured households themselves.

Since the treatment was not randomly assigned, simply comparing insured to non-insured households will lead to selection bias as the two types of households may be systematically different. For example, insured households may be less healthy and more likely to seek health care than non-insured households which could bias insurance effects downwards.

The second part of our empirical strategy addresses this by estimating the impact of the program on only those who enrolled in the treatment area by comparing them to similar individuals in the control group. We operationalize this second approach using propensity score weighting; applying weights on the basis of the propensity score – i.e., the probability of being insured in the treatment area and predicting this probability for the households in the control area as well [62, 63]. This estimate gives the average treatment effect on the treated (insured) population – the ATET-PS, as follows.

We first calculated the propensity scores by fitting a logit model to estimate the likelihood *P*_*i*_ of household *i* being enrolled in the insurance program for the treatment area only.

2 $${\varvec{l}}ogit \left({P}_{i} \right)={{ X}_{i}}^{^{\prime}}\rho +{\varepsilon }_{i}$$

The included baseline characteristics *X*_*i*_ in our specification are as listed in Table 1 and include baseline age, gender and education of household head, household size, household location (town or village), distance to nearest program (KSHIP) clinic, annual non-medical household consumption (based on weekly food expenditures, and monthly and annual non-food expenditures aggregated to the annual level), annual household health expenditure at baseline, and indicator variables for whether household has good toilet, whether household has good water, whether all household members can carry out daily activities without any difficulty, whether anyone in the household had a chronic disease in the past year, whether anyone in the household had an acute illness or injury in the past year and measures of CHE at baseline. This model was estimated for insurance status in the treatment area in 2011 and in 2013 separately. Next, the coefficients were used to predict the propensity scores (pscore) for the sample of insured households in the treatment area and all households in the control area in 2011 and in 2013, respectively. These propensity scores were then applied as inverse probability weights in the original DiD regression model specification, with a weight of 1 for insured respondents in the treatment area and a weight of ‘pscore/1-pscore’ for respondents in the control area [62, 64, 65]. We conducted the estimations in separate regressions comparing outcomes in 2011 versus 2009, and then outcomes in 2013 versus 2009.

Furthermore, we investigated heterogeneity in impact by baseline wealth terciles – poor, middle and rich. The wealth terciles were constructed based on the first loading of a principal component analysis of 30 dwelling characteristics and asset ownership indicators at baseline. We also investigated heterogeneous treatment effects by baseline health status for households with and without a chronically ill household member at baseline.

We conducted several sensitivity analyses to check the robustness of our findings. We implemented an instrumental variables (IV) approach to estimate the ATET-IV by using treatment area as an instrument for household insurance status. In addition, we ran the ITT regressions using different thresholds for the numerator of our CHE proxies to test the sensitivity of our impact estimates to changes in the threshold value – in particular, we compared the 10% of household consumption with 5%, 15%, and 20% thresholds; and the 40% of CTP with 10%, 20%, and 30% thresholds. We also ran probit regressions as a non-linear estimation approach to assess the robustness of our estimates considering the relatively low prevalence of the outcome.

These analyses focus on the balanced panel across the three survey waves of 1,039 households. The attrition rate between the full sample at baseline and the balanced panel after four years was 28.9%. Households in the treatment area were less likely to attrit compared to households in the control area (Additional file 1: Table 1). However, across most baseline characteristics, there was no significant difference between households who attrited in the treatment area compared to the control area, limiting concerns of selective attrition. The two exceptions were urban and higher educated households that were more likely to attrit in the treatment compared to the control group. We take these variables into account in the ITT through the household-fixed effects and by including them in the propensity score model for the ATET. We also investigate the robustness of our findings to the inclusion of attrition weights in the regressions. All analyses were conducted in Stata/MP 15 and R.

## Results

### Descriptive Data

Table 1 shows the means of the baseline characteristics for the total balanced panel in Column (1) and compares the means between the treatment and control areas in Columns (2)-(4). (See Additional file 1: Table 2 for the characteristics of the total unbalanced sample at baseline). Household heads were on average 52 years old, 22.1% were female, and 79.6% were married. The average household consisted of 4.4 members. Slightly more than half of the household heads (55.9%) had completed primary education, and most (93.0%) had worked for income in the past year. Annual non-medical household consumption was approximately 561,000 Naira after correcting for inflation; with 41% of households living below the absolute poverty line in 2009–2010 [51]. About 80% of households had access to good quality drinking water but only 8.1% had access to a good quality toilet. About half of them (51.9%) were living in an urban area. The average distance to the nearest program facility (or potential program facility in the control area) was 3.2 km, while the average distance to any facility was 1.2 km. In the total sample, 87.3% of individuals could perform their daily activities without any difficulties. This translates into 31.1% of households having all household members being able to perform daily activities without difficulty. Whereas 6.9% of individuals were chronically ill, 21.8% of households had at least one member with a chronic disease. Finally, 40.0% of households had experienced at least one acute illness or injury in the past year.

Household heads in the treatment area were older, more likely to be male, and less educated compared to those in the control area. Marital status and employment of the head, as well as household size did not differ across treatment and control groups. Annual non-medical household expenditures as well as annual health spending were also similar. However, the wealth index based on dwelling characteristics and amenities (such as toilet and drinking water) indicated that the control households were on average somewhat better off. Differences across the health-related variables were not statistically significant, except for the ability to do daily activities, which was higher in the control households.

Table 1 Columns (5–10) show that households in the treatment area who were insured in 2011 or 2013 were more likely to have a male and married household head compared to those who were uninsured in the treatment area. They were also larger (2013 only), better educated (2011 only), wealthier, and more likely to live in town and close to a clinic. Finally, insurance status was associated with worse health, especially in 2013; insured households were more likely to have a household member with a chronic or acute illness in the past year, or who was not able to do daily activities without difficulty.

Table 2 describes the outcome measure, catastrophic health expenditures (CHE), using the two definitions, for the full sample, the treatment area, and the control area, in each of the survey rounds. Using the 10% threshold of household consumption, 1.9% of households experienced CHE at baseline in 2009, with no significant difference between the treatment and control areas. The likelihood of experiencing CHE increased to 3.0% on average in 2011 and 2013, with households residing in the control area having significantly higher CHE occurrence in 2011 at 5.7% compared to households in the treatment area at 1.5%. In 2013, the mean occurrence of CHE in control areas was still larger than in treatment areas but the difference was no longer statistically significant.

**Table 2:** Means of CHE & Health Insurance Variables (Balanced panel)

		**2009**				**2011**				**2013**			
		Total	Control	Treatment	*P*-value of Diff. of Means	Total	Control	Treatment	Mean Diff & *p*-value	Total	Control	Treatment	*P*-value of Diff. of Means
**CHE **													
***Number of observations***		1039	367	672	1039	1039	367	672	1039	1039	367	672	1039
Catastrophic health exp (10% threshold)	Mean	0.019	0.019	0.019	0.976	0.030	0.057	0.015	0.000***	0.030	0.035	0.027	0.435
	SD	(0.137)	(0.137)	(0.138)		(0.170)	(0.233)	(0.121)		(0.170)	(0.185)	(0.162)	
Catastrophic health exp (> = 40% of CTP)	Mean	0.013	0.008	0.015	0.353	0.019	0.019	0.019	0.976	0.034	0.035	0.033	0.819
	SD	(0.111)	(0.090)	(0.121)		(0.137)	(0.137)	(0.138)		(0.181)	(0.185)	(0.178)	
**Health** **Insurance status**													
Insured (% of individuals)	Mean	0.002	0.002	0.002	0.927	0.205	0.001	0.319	0.000***	0.205	0.001	0.319	0.000***
	SD	(0.048)	(0.049)	(0.048)		(0.404)	(0.029)	(0.466)		(0.404)	(0.028)	(0.466)	
Insured (% of households)	Mean	0.010	0.008	0.010	0.724	0.295	0.003	0.454	0.000***	0.333	0.005	0.512	0.000***
	SD	(0.098)	(0.090)	(0.102)		(0.456)	(0.052)	(0.498)		(0.472)	(0.074)	(0.500)	

Based on the balanced panel of 1,039 households and 3,450 individuals

^*^ p < 0.1; ** p < 0.05; *** p < *0.01.* Standard deviation in parentheses. Standard errors for Diff of means clustered at EA level

Treatment and control indicate households living in the treatment and the control area at baseline, respectively

Catastrophic health exp (10% threshold): indicator variable coded as 1 if out-of-pocket health spending is greater than 10% of annual household consumption

Catastrophic health exp (40% of CTP): indicator variable coded as 1 if out-of-pocket health spending is greater than 40% of household capacity to pay

Insured for households is an indicator variable coded as 1 if a respondent is insured in the health insurance program. At the household level, Insured is coded as 1 if at least one person in the household is enrolled in the health insurance

Using the 40% CTP threshold, 1.3% of households experienced CHE at baseline, both in the treatment and the control area. This increased to 1.9% in 2011 and to 3.4% in 2013. Using this measure, the difference in CHE occurrence between treatment and control areas at baseline and follow-up periods was not statistically significant.

Table 2 also presents the means of health insurance status across the three time periods. Insurance coverage was very low at baseline in both treatment and control areas, with 1.0% of households having at least one insured member. Overall, 0.2% of individuals were enrolled in health insurance in 2009. During the follow-up periods, the proportion of insured individuals remained negligible at 0.1% in the control group while the proportion of insured individuals in the treatment area increased to 31.9% both in 2011 and 2013. The proportion of households with at least one insured household member in the treatment area increased from 1.0% at baseline to 45.4% in 2011 and 51.2% in 2013.

### Intention-to-treat estimates

Table 3 shows the results for the ITT estimates for both the short-term (2009–2011), soon after introduction of the scheme, and for the longer-term (2009–2013), four years later. Panel A shows the estimates when measuring the CHE outcome as OOPs exceeding 10% of household consumption, while Panel B presents the estimates when measuring CHE as OOPs exceeding 40% of capacity to pay. Columns (1) and (2) show the impact estimates for the total population without and with controls, respectively.

**Table 3:** Impact of health insurance on CHE for full sample, by wealth terciles and by chronic illness status [(Intention-to-treat estimates 2011 and 2013)]

	(1)	(2)	(3)	(4)	(5)	(6)	(7)
	**Full sample** **-no controls**	**Full sample-controls**	**Poor**	**Middle**	**Rich**	**Chronic**	**Non-Chronic**
**Panel A: CHE (> 10% of household consumption)**							
ITT 2011	-0.043**	-0.043**	-0.072***	-0.032	-0.036	-0.094***	-0.029
	(0.017)	(0.017)	(0.025)	(0.027)	(0.025)	(0.033)	(0.018)
ITT 2013	-0.009	-0.009	-0.015	-0.012	-0.012	-0.025	-0.005
	(0.015)	(0.015)	(0.023)	(0.025)	(0.022)	(0.034)	(0.016)
N	3117	3096	1034	1032	1030	678	2418
Adj. R-sq	0.005	0.006	0.013	0.008	0.002	0.007	0.004
**Panel B: CHE (> = 40% of capacity to pay)**							
ITT 2011	-0.006	-0.007	-0.011	-0.006	-0.012	-0.043	0.004
	(0.011)	(0.011)	(0.022)	(0.015)	(0.014)	(0.030)	(0.012)
ITT 2013	-0.009	-0.011	-0.058*	-0.016	0.006	-0.024	-0.007
	(0.013)	(0.013)	(0.035)	(0.021)	(0.014)	(0.032)	(0.013)
N	3117	3096	1034	1032	1030	678	2418
Adj. R-sq	0.002	0.020	0.032	0.006	0.003	0.022	0.017

Standard errors clustered at EA level (in parentheses)

^*^ *p* < 0.10; ** *p* < 0.05; *** *p* < 0.01

Based on the balanced panel of 1,039 households

Covariates included in the ITT regressions are gender and marital status of the household head, and household size

Residing in the treatment area is significantly associated with a 4.3 percentage points decrease in short-term CHE occurrence using the 10% of consumption threshold. This impact decreases to 0.9 percentage points and becomes insignificant four years after program introduction. Neither the short- nor the longer-term impact estimates of CHE based on household CTP are statistically significant, although signs are negative as expected.

To investigate whether our results are sensitive to attrition, Additional file: Table 3 shows the ITT estimates weighted for attrition. The findings are robust to the weighting procedure. The estimates in Panel A slightly increase in size but remain of the same order of magnitude, and their precision increases. The 40% of CTP estimates in Panel B remain negative but small and not significant.

We further examine the sensitivity of our findings to the thresholds used (i.e. the numerators in the two measures). Results are shown in Additional file: Table 3. As expected, the impact on the probability of CHE monotonically decreases as the threshold becomes larger. Columns (1)-(5) describe the estimates for thresholds from 5 to 25% of household consumption with stepwise 5%-increments. The short-term impact estimate loses its significance only at the 25% threshold. Columns (6)-(9) show the estimates for thresholds from 10 to 40% of CTP with stepwise 10%-increments. The short-term impact is statistically significant only at the 10% threshold with an estimate of 7.5 percentage points. Longer-term impacts remain insignificant for both measures, irrespective of the threshold used. These findings suggest that both the significantly negative short-term estimates and the non-significant longer-term estimates are robust, and that the 40% CTP threshold – as commonly used in the literature – may be too conservative, lacking distinctiveness for our sample.

### Average treatment effect on the treated (ATET) estimates

**Fig. 1:**
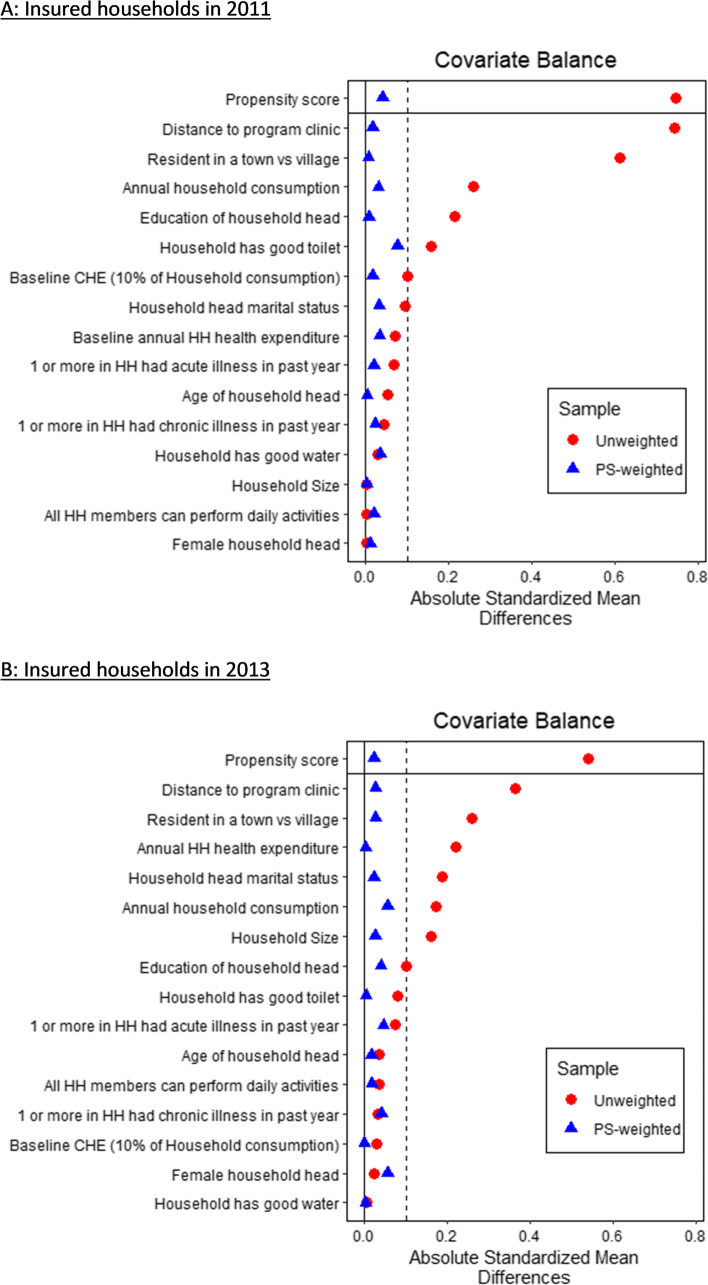
Absolute standardized differences in unweighted and weighted samples [CHE (10% of household consumption)]. Results based on separate logistic regressions of insurance status in the follow-on years—2011 and 2013 on the explanatory variables shown in the figure. Balance is assessed by comparing the means of unweighted and weighted treatment and control in the full balanced sample using absolute standardized differences. Balance is said to be achieved if absolute standardized difference is less than the 0.1 threshold (stripped vertical line)

**Fig. 2:**
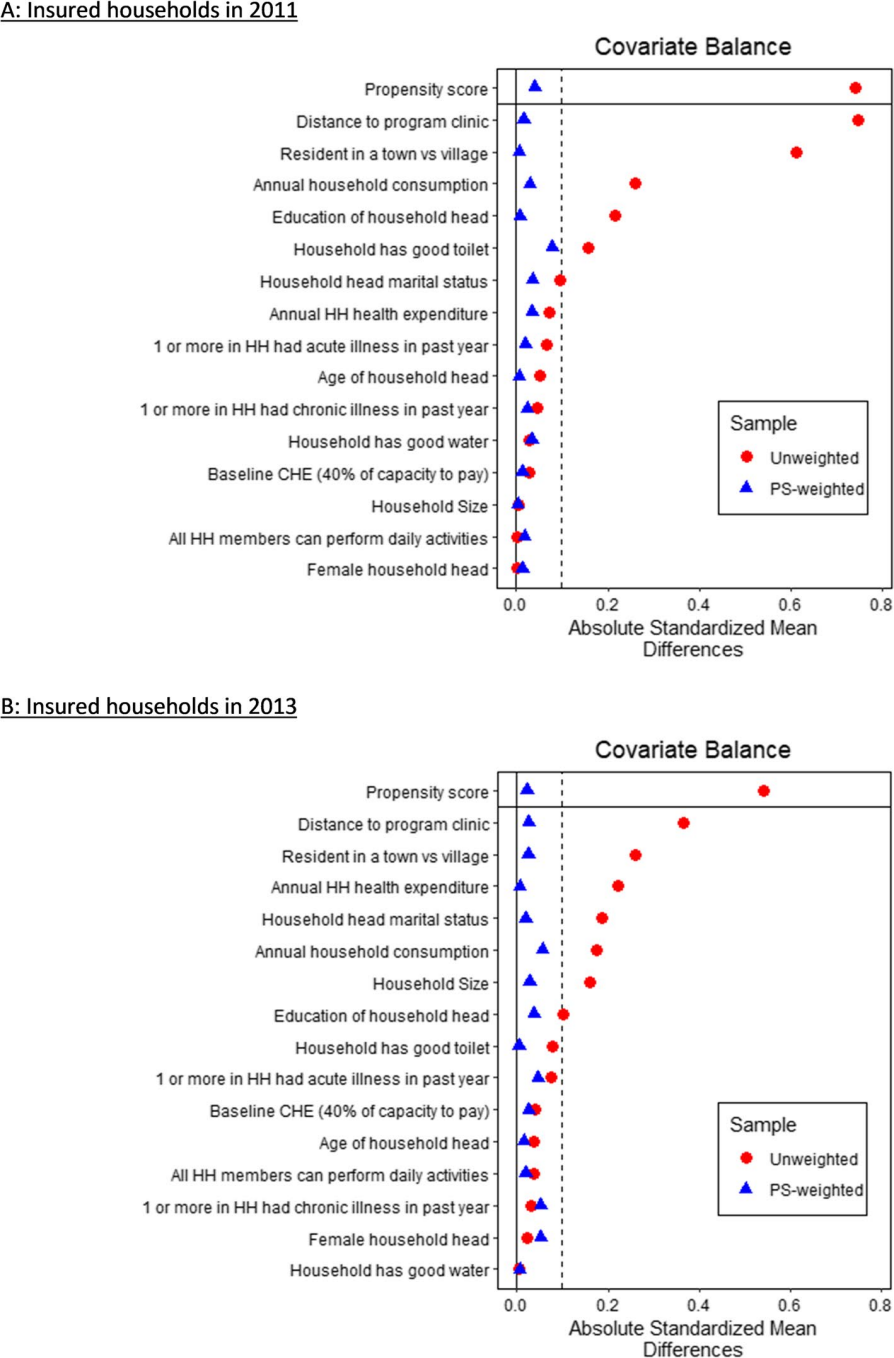
Absolute standardized differences in unweighted and weighted samples [CHE (40% of capacity to pay)]. Results based on separate logistic regressions of insurance status in the follow-on years—2011 and 2013 on the explanatory variables shown in the figure. Balance is assessed by comparing the means of unweighted and weighted treatment and control in the full balanced sample using absolute standardized differences. Balance is said to be achieved if absolute standardized difference is less than the 0.1 threshold (stripped vertical line)

Estimates of the logit model for generating propensity scores indicate that the probability of being insured is higher for households closer to a program health facility in both 2011 and 2013. In addition, wealthier households in terms of quality toilet ownership are more likely to be enrolled in 2011 while households with married and female heads and higher health spending are more likely to be enrolled in 2013 (Additional file: Table 4). Figures 1 & 2 show absolute standardized mean differences between treatment and control areas after propensity score weighting, indicating there are no residual systematic differences in observed baseline characteristics between treated and control observations in the sample weighted by the estimated inverse probability weights.

Table 4 Panels A and B show the ATET estimates in the short- and longer-term for CHE measured as 10% of household consumption and 40% of CTP, respectively. Columns (1) and (2) show the impact estimates for the total population without and with controls, respectively. Our results show a 5.7 percentage points reduction in short-term occurrence of CHE using the 10% threshold, when controlling for baseline controls (Panel A). The longer-term effect is not statistically significant, although the estimate is negative. Using the CTP measure, we find no significant effect of health insurance on CHE occurrence in the short-term, while the longer-term estimate suggests a decrease in CHE of 2.7 percentage points for the insured (Panel B)

**Table 4:** Impact of health insurance on CHE for full sample, by wealth terciles and by chronic illness status [(Average treatment effect on the treated estimates 2011 and 2013)]

	(1)	(2)	(3)	(4)	(5)	(6)	(7)
	**Full sample** **-no controls**	**Full sample-controls**	**Poor**	**Middle**	**Rich**	**Chronic**	**Non- Chronic**
**Panel A: CHE (> 10% of household consumption)**							
ATET 2011	-0.021	-0.057**	-0.062**	-0.047	-0.049	-0.076**	-0.035
	(0.014)	(0.025)	(0.030)	(0.035)	(0.052)	(0.036)	(0.028)
N	667	667	165	217	273	122	521
Adj. R-sq	0.001	0.014	-0.035	0.065	0.0009	0	0.019
ATET 2013	-0.009	-0.019	0.008	-0.054**	-0.034	-0.02	0.009
	(0.018)	(0.019)	(0.027)	(0.024)	(0.041)	(0.045)	(0.026)
N	709	709	197	218	282	132	553
Adj. R-sq	-0.001	0.019	0.019	0.081	0.177	0.014	0.089
**Panel B: CHE (> = 40% of capacity to pay)**							
ATET 2011	-0.006	-0.026	-0.036	-0.004	-0.024	-0.071	0.003
	(0.011	(0.018)	(0.038)	(0.016)	(0.032)	(0.048)	(0.017)
N	667	667	165	217	273	122	521
Adj. R-sq	0.001	0.018	-0.012	0.068	0.054	0.023	0.002
ATET 2013	-0.001	-0.027*	-0.065	-0.056**	0.025	0.029	-0.037**
	(0.014)	(0.016)	(0.046)	(0.025)	(0.029)	(0.035)	(0.015)
N	709	709	197	218	282	132	550
Adj. R-sq	0.001	0.026	0.019	0.127	0.244	0.03	0.025

Standard errors clustered at EA level (in parentheses); * *p* < 0.10; ** *p* < 0.05; *** *p* < 0.01

Based on the balanced panel of 1,039 households. Covariates included in regressions are baseline values for gender, marital status and education of household head, household size, urban, distance to program health facity, household consumption excluding health expenditure, quality of toilet and water, household health expenditure. Propensity score weighting applied as inverse probability of treatment weights. Wealth index: These are calculated as baseline wealth terciles and were constructed based on the first loading of a principal component analysis of 30 dwelling characteristics and asset ownership indicators measured at baseline

Table 5 repeats the ATET analyses using an IV estimator instead of propensity score weighting. Panel A again presents the results for the 10% of household consumption measure, reinforcing the propensity score findings. In the short-term, the program is estimated to reduce the probability of CHE with 10.2 percentage points for insured households in the treatment area; longer-term effects are again not significant. The 40% of CTP measure (Panel B) yields non-significant impact estimates close to zero in both the short- and longer-term. Additional file: Table 5 shows that the ATET-IV results do not change when observations are weighted for attrition.

**Table 5:** Impact of health insurance on CHE for full sample, by wealth terciles and by chronic illness status, [(Instrumental Variables Approach 2011 and 2013)]

	(1)	(2)	(3)	(4)	(5)	(6)	(7)
	**Full sample** **-no controls**	**Full sample-controls**	**Poor**	**Middle**	**Rich**	**Chronic**	**Non- Chronic**
**Panel A: CHE (> 10% of household consumption)**							
Household Insured 2011	-0.095***	-0.102***	-0.172**	-0.080	-0.080**	-0.157**	-0.089***
	(0.029)	(0.030)	(0.087)	(0.049)	(0.034)	(0.069)	(0.033)
* Controls*	*No*	*Yes*	*Yes*	*Yes*	*Yes*	*Yes*	*Yes*
N	1036	1036	347	345	344	226	810
Household Insured 2013	-0.017	-0.016	0.004	-0.037	-0.026	0.022	-0.025
	(0.023)	(0.022)	(0.054)	(0.040)	(0.022)	(0.051)	(0.025)
* Controls*	*No*	*Yes*	*Yes*	*Yes*	*Yes*	*Yes*	*Yes*
N	1039	1039	347	346	346	227	812
**Panel B: CHE (> = 40% of capacity to pay)**							
Household Insured 2011	0.000	0.000	0.059	-0.009	-0.043**	-0.008	0.003
	(0.020)	(0.021)	(0.070)	(0.020)	(0.021)	(0.061)	(0.021)
* Controls*	*No*	*Yes*	*Yes*	*Yes*	*Yes*	*Yes*	*Yes*
N	1036	1036	347	345	344	226	810
Household Insured 2013	-0.005	0.000	-0.040	-0.027	-0.006	0.041	-0.007
	(0.023)	(0.023)	(0.073)	(0.038)	(0.014)	(0.052)	(0.025)
* Controls*	*No*	*Yes*	*Yes*	*Yes*	*Yes*	*Yes*	*Yes*
N	1039	1039	347	346	346	227	812

Standard errors clustered at EA level (in parentheses)

^*^ *p* < 0.10; ** *p* < 0.05; *** *p* < 0.01

Based on the balanced panel of 1,039 households

Covariates included in regressions are gender and marital status of household head, household size

### Heterogeneity analyses

The results of the heterogeneity analyses by baseline household wealth terciles are shown in Columns (3)-(5) of Tables 3–5. The results show significant heterogeneity in the ITT impact estimates by baseline wealth (Table 3). Using the 10% consumption threshold in Panel A, among those in the poorest wealth tercile, there is a 7.2 percentage points reduction in CHE occurrence in the short-term while estimates for the other wealth terciles are lower in magnitude and statistically insignificant. No statistically significant effect was found in the longer-term for any of the wealth terciles. Using the 40% CTP measure, we do not find significant heterogeneity by wealth in the short-term but a significant longer-term reduction of 5.8 percentage points in CHE occurrence among the poorest wealth tercile compared to negligible impact estimates for the wealthier terciles.

Heterogeneous ATET impact estimates in Table 4 Columns (3)-(5) indicate that the probability of experiencing CHE in the short term decreases by 6.2 percentage points for households in the poorest wealth tercile using the 10% threshold (Panel A). Short-term impacts on the middle and rich terciles are smaller in magnitude and not significant, like the ITT estimates. In the longer-term, the middle tercile appear to benefit most from the insurance scheme with a significant reduction in CHE of 5.4 percentage points. The ATET findings using the 40% CTP threshold (Panel B) are similar, although the short-term impact on the poorest tercile is not statistically significant.

The ATET-IV robustness check in Table 5 Columns (3)-(5) confirms that the short-term impact of insurance is largest for the poorest tercile when using the 10% of consumption threshold (Panel A) with an estimated reduction in the probability of CHE of 17.9 percentage points. In the longer-term, significance is lost while the estimate remains largest for the middle tercile. Interestingly, the short-term impact on the richest tercile is statistically significant for both measures when estimating the ATET-IV, as shown in Column (5) Panels A and B.

We further examine heterogeneity in the estimates among households with different levels of baseline health status. Specifically, in Tables 3–5 Columns (6) and (7) we compare households with at least one member with a chronic illness at baseline (n = 226) with households without a chronic illness (n = 813). The relatively small sample size of chronically ill households warrants some caution in the interpretation of the results. Our findings in Panel A show that households with a member who was chronically ill at baseline are at increased risk of experiencing CHE in 2011, and that access to the insurance program decreases this risk. The findings suggest an 9.4 percentage points reduction in short-term CHE occurrence among households with a chronic illness compared to a 2.9 percentage points reduction for households without a chronically ill member (Table 3, Columns 6 and 7, respectively). The longer-term impact is also larger for chronically ill households at 2.5 percentage points, although the coefficient is imprecisely estimated potentially due to the relatively small sample size. Neither the short- nor the longer-term ITT impact estimates among households with a chronic illness are statistically significant when using 40% of CTP measure (Table 3 Panel B), although the coefficient remains larger for households with a chronically ill member.

The ATET findings in Tables 4 and 5, estimated with propensity score weighting and IV, respectively, also show larger point estimates for the chronically ill than the non-chronically ill with reductions in CHE of 7.6 and 15.7 percentage points in the short-term, respectively (Panels A). Using the 40% of CTP measure (Panel B) yields a similar albeit mostly non-significant pattern in both the short- and longer-term. Finally, our findings are robust to non-linear estimation as shown for ITT probit regressions in Additional file: Table 6.

## Discussion

Households in low- and middle-income countries like Nigeria are at significant risk of financial catastrophe due to their high levels of out-of-pocket payments. One of the potential benefits of health insurance is to protect households from such exposure. This study examines the impact of a subsidized voluntary health insurance intervention in Kwara state, Nigeria, on financial protection—proxied by the reduction in CHE occurrence, both in the short-term two years after the introduction of the program, and in the longer-term after four years of program implementation. When measuring CHE as OOPs exceeding 10% of household consumption, living in the program area is associated with a significant short-term reduction in the probability of CHE of 4.7 percentage points. The reduction is equivalent to a 73% decrease compared to the mean in the control group at short-term follow-up. In the longer-term however, impacts are diluted and are no longer statistically significant. These findings are in line with a previous impact evaluation of the insurance program that found a 51% decrease in out-of-pocket health spending after two years, while OOPs returned to pre-program levels four years after the introduction of the scheme [53]. Positive impacts on healthcare utilization remained high over the entire period at 25.2 and 20.4 percentage points after two and four years, respectively.

The ITT estimates represent the impact of living in the program area, whether insured or not. They are useful in understanding the impact of a program at the population level, but they do not provide estimates for those households who actually took up insurance. The ATET-PS estimates instead use propensity score weighting to compare insured households with similar households in the control area, correcting for systematic differences between insured and uninsured households based on observable baseline characteristics. Our ATET-PS findings suggest that the health insurance program reduced the risk of catastrophic health expenditures significantly with 5.7 percentage points for households who enrolled in insurance shortly after the introduction of the scheme. ATET-IV estimates based on an instrumental variables analysis suggest a reduction of up to 10.2 percentage points for the insured. Again, the impacts fade out four years after the program was introduced, regardless of the method used. Whereas the coefficients remain negative, the results are less precise and no longer significant.

However, the definition used to construct CHE matters: We observe a more consistent pattern of positive effects with the 10% of household consumption measure compared to 40% of CTP. This is similar to findings by Axelson et. Al. (2009) and Giedion and Uribe (2010) who report varying magnitudes of effects depending on the thresholds of CHE used [38, 66]. Both studies did not find significant reductions at the 40% CTP threshold. There is also some skepticism in the literature that the CTP approach may not necessarily indicate whether expenses are large enough to undermine a household’s ability to purchase nonmedical necessities. This could explain our lack of significant findings compared to using the 10% threshold of consumption [67]. In line with this interpretation, our sensitivity analyses show that the impact findings are robust to a large range of thresholds when using household consumption as denominator, while the estimates are very sensitive to the threshold when using CTP as denominator and not significant except for the lowest (least conservative) threshold level.

Our findings on the short-term reduction of CHE are consistent with most existing studies in SSA and elsewhere [30, 33–35]. We find no sustained reduction in CHE occurrence several years after insurance roll-out, similar to findings by Knaul et al. (2018) who also report no significant effects after eight years in their study of *the Seguro Popular* in Mexico. Our finding of null effects in the long-term could be due to insured households adjusting their behaviour over time. If the program stimulates households to increase health care utilization, this could induce them to also incur more non-covered costs such as for medicines or surgeries, as suggested by findings from Bernal et al. (2017), Wagstaff and Lindelow (2008) and Sparrow et al. (2013) [32, 43, 44]. Alternatively, in the longer-term, insured households may use not only the program facilities covered by the scheme but also revert to non-program facilities due to travel considerations (program facilities are on average three times farther than non-program facilities) or quality motives [68], where they still run a risk of incurring high OOPs. This highlights the need to expand accreditation to more facilities by investing in necessary upgrades for qualification as well as paying attention to continuous quality improvement in participating health facilities.

These mechanisms might also shed some light on our unexpected findings that the ITT estimates are larger than the ATET-PS estimates for some sub-samples. A potential explanation is as follows. First, this must mean that the uninsured in treatment areas also benefit from the new insurance scheme in terms of reduced OOPs. One mechanism might run through the enhanced quality of facilities – indeed, the facility upgrade attracted both insured and uninsured individuals to seek more care of better quality [53]. This in turn could have reduced the delay in seeking care, the length of illness spells, the severity of the illness and/or the need for follow-up treatments – all of which might translate into decreased OOPs, irrespective of insurance coverage. Second, part of the quality-related reductions in OOPs might have cancelled out for the insured if their enhanced access led to more treatments and services that were not fully covered by the scheme (such as medications or specialized services). In that case, part of their increased utilization may have led to out-of-pocket payments, as described in the literature above. This would affect our CHE estimates especially if a relatively large share of the insured households were close to the CHE threshold to begin with. This seems a reasonable assumption, given that insurance is voluntary and subject to selective uptake.

Our study further explores heterogeneity in impact of health insurance and finds substantial evidence that insurance is particularly beneficial for the poorest households in the short-term, but not the longer-term. The reduction in the risk of catastrophic health expenditures among insured households in the poorest tercile is almost double the effect on the average insured household. We found no significant evidence of similar reductions in the middle and rich wealth terciles. This is consistent with Saksena et al. (2006) who documented evidence of a larger reduction in CHE among the insured poor than the insured rich in Kenya, and Grogger et al. (2015) who also reported reduced CHE especially in rural areas in Mexico where the population is relatively poor.

The lack of longer-term impacts for the poor is particularly relevant considering the paucity of evidence in this regard. It highlights the need for sustained efforts to keep health insurance effective in protecting the most vulnerable households. Future research is needed to understand the reasons why impacts were fading out over time.

Furthermore, we find particularly pronounced reductions in the short-term probability of CHE among households with at least one member with a chronic illness. The ITT, ATET-PS and ATET-IV estimates are 9.4 pp, 7.6 pp and 15.7 pp, respectively. Longer-term effects are imprecisely estimated. This illustrates the protective effect of health insurance on those households with higher needs for health care. The strong impacts on the chronically ill are highly relevant given their increased vulnerability for suffering from CHE [57, 69], and in view of the continuously increasing contribution of chronic illnesses to the disease burden in low- and middle-income countries.

This study has several limitations. First, insurance status is measured at the individual level, but not all households decide to enroll all their household members. We define a household as being insured if at least one member has health insurance. While this is a commonly used construct for household-level insurance status when full household coverage is not mandated, it may lead to an underestimation of the impact of health insurance. Also, the data on health expenditures were collected at the annual and household level; as a result, they could not directly be linked to the insurance status of the ill individual at the time of health care utilization when costs were incurred. Future research would benefit from more detailed individual-level data on health expenditures and timing of illnesses and injuries. In addition, enrolment is voluntary and may hence be selective. Although the ATET-PS estimates control for selection on observables, they do not correct for selection on time-varying unobservable characteristics. These results should hence be interpreted with caution. Finally, the intervention was not implemented randomly across treatment and control areas. Despite the careful selection of the areas to be as comparable as possible, we observe some differences in baseline characteristics between the two areas – albeit not with respect to our key outcome variable. To the extent that both areas experienced the same trends over time – supported by their geographical proximity, and comparability in terms of socio-economic and health systems – the differences-in-differences methodology, the propensity score weighted regression estimates and the instrumental variable analyses will adjust for observed and unobserved differences between the two groups. Despite these limitations, the consistently negative short-term impact estimates provide assurance that the findings are robust, even if their statistical significance varies across CHE thresholds and specifications.

## Conclusions

This study provides empirical support for continued investments in health insurance reform and scale-up such that households, including the poor and the chronically ill, who are most at risk of experiencing financial catastrophe due to uninsured ill health, are protected. Our results highlight that subsidized voluntary or social health insurance schemes targeted at the poor can serve as an important equity-enhancing instrument in the society, support the pursuit of universal health coverage and prevent the most vulnerable households from falling further into poverty. However, the significant impacts in the first two years after program roll-out were not sustained in the longer-term. This calls for action to ensure that insurance schemes remain effective in protecting households from financial risk, even as households adjust their care-seeking behavior over time.

## Supplementary information

**Additional file 1.**

## Data Availability

The datasets used and analyzed during the current study are available from the corresponding author on reasonable request.

## Abbreviations

OOPs: Out-of-pocket expenditures
CHE: Catastrophic health expenditure
LMICs: Low- and middle-income countries
UHC: Universal health coverage
SSA: Sub-Saharan Africa
EA: Enumeration areas
KSHIP: The Kwara State Health Insurance Program
ITT: Intention-to-treat
DiD: Difference-in-differences
ATET: Average treatment effect on the treated
