# Supplementary material for: Short and longer-term impacts of health insurance on catastrophic health expenditures in Kwara State, Nigeria
Source: BMC Health Serv Res. 2022 Dec 20;22:1557. doi: 10.1186/s12913-022-08917-z (PMC9764477; doi:10.1186/s12913-022-08917-z)
Supplement: Supplementary file 1 — Additional file 1. [file 12913_2022_8917_MOESM1_ESM.pdf]

Short- and longer-term impacts of health insurance on catastrophic health expenditures in Kwara State, Nigeria

Additional File 1

Table 1: Household Attrition Analysis

	(1)	(2)	(3)	(4)	(5)	(6)	(7)	(8)	(9)	(10)	(11)	(12)	(13)
		Household head is Female	Urban	HH head: At least primary school education	Household size(>median)	Wealth index: poor	Wealth index: middle	Wealth index: rich	Good quality toilet	Good quality drinking water	Dummy: All HH members can do daily activities without difficulty	Dummy: at least 1 HH member has a chronic disease	Dummy: at least 1 HH member had an acute illness/injury in past 12 months
Treatment	-0.110***	-0.096*	-0.200***	-0.212***	-0.074	-0.114***	-0.092**	-0.145***	-0.129***	-0.181*	-0.110*	-0.110**	-0.098**
	(0.029)	(0.042)	(0.034)	(0.046)	(0.040)	(0.033)	(0.033)	(0.036)	(0.032)	(0.073)	(0.049)	(0.033)	(0.037)
Variable		0.086	-0.143***	-0.170***	-0.146***	0.167***	0.017	-0.173***	-0.109*	-0.015	-0.006	-0.072	0.004
		(0.061)	(0.034)	(0.049)	(0.040)	(0.043)	(0.052)	(0.046)	(0.050)	(0.071)	(0.050)	(0.049)	(0.040)
**Variable # Treatment**		-0.029	0.175***	0.164**	-0.077	-0.054	-0.056	0.049	0.093	0.089	-0.002	-0.011	-0.034
		(0.074)	(0.051)	(0.059)	(0.050)	(0.054)	(0.061)	(0.057)	(0.146)	(0.079)	(0.059)	(0.060)	(0.052)
Constant	0.357***	0.331***	0.431***	0.462***	0.441***	0.305***	0.353***	0.417***	0.377***	0.371***	0.362***	0.373***	0.356***
	(0.021)	(0.034)	(0.027)	(0.038)	(0.028)	(0.023)	(0.024)	(0.028)	(0.025)	(0.068)	(0.040)	(0.025)	(0.027)
Observations	1462	1462	1462	1462	1462	1462	1462	1462	1462	1462	1462	1462	1462
Adj R-squared	0.013	0.017	0.023	0.025	0.059	0.036	0.014	0.033	0.015	0.015	0.012	0.017	0.013

Standard errors in parentheses and clustered at EA level
=* p<0.05, ** p<0.01, *** p<0.001

Linear regression of the probability of households to attrit after baseline on a treatment indicator (i.e., living in a treatment area at baseline), household baseline characteristics as indicated in the top row, and an interaction term between treatment and the characteristics which captures differential attrition between treatment and control households.

Wealth index: These are calculated as wealth terciles and were constructed based on the first loading of a principal component analysis of 30 dwelling characteristics and asset ownership indicators.

Table 2: Baseline Household Characteristics (Unbalanced panel, 2009)

	2009 Full	2009 Control	2009 Treatment	Mean Difference and p-values
Demographic characteristics				
Age of Household head	52.61	49.83	54.39	4.526***
Household head is Female	0.243	0.306	0.203	-0.104***
Dummy: household head is married	0.763	0.755	0.768	0.012
Household size	4.084	4.114	4.065	-0.052
Socio-economic characteristics				
HH head: At least primary school education	0.540	0.613	0.493	-0.119**
Dummy: household head has worked in past year	0.920	0.915	0.924	0.008
Annual non-medical consumption of household (in Naira)	532,809	577,187	504,387	-72,511*
Wealth Index (1-3)	1.999	2.193	1.876	0.317***
Wealth Index=Poor	0.408	0.303	0.475	0.173***
Wealth Index=Middle	0.336	0.352	0.326	-0.027
Wealth Index=Rich	0.256	0.345	0.198	-0.146***
Good quality toilet	0.0779	0.177	0.0146	-0.162***
Good quality drinking water	0.821	0.891	0.776	-0.116***
Urban: Household lives in a town vs village	0.506	0.517	0.500	-0.017
Health-related characteristics				
Distance (km) to nearest HCHP clinic	3.269	3.906	2.862	-1.041*
Distance (km) to nearest clinic (all 79 clinics)	1.205	1.263	1.169	-0.095
Dummy: All HH members can do daily activities without difficulty	0.692	0.797	0.626	-0.170***
Dummy: at least 1 HH member has a chronic disease	0.202	0.215	0.193	-0.022
Dummy: at least 1 HH member had an acute illness/injury in past 12 months	0.398	0.448	0.365	-0.083**
HH annual health expenditures (excl. premium)	4891	4971	4836	-167.2
Dummy at least one person in household is insured	0.010	0.011	0.009	0.0016
**Observations**	**1463**	**571**	**892**	**1463**

* p<0.1; ** p<0.05; *** p<0.01
Standard errors in parentheses, corrected for clustering at EA level.
Based on the full unbalanced panel of 1,463 households at baseline.

Table 3: Impact of health insurance on CHE for full sample, by wealth terciles and by chronic illness status with Attrition Weights [(Intention to treat estimates 2011 and 2013)]

	(1) **Full sample -no controls**	(2) **Full sample-controls**	(3) **poor**	(4) **middle**	(5) **rich**	(6) **chronic**	(7) **Non-chronic**
**Panel A: CHE (> 10% of household consumption)**							
ITT 2011	-0.047***	-0.047***	-0.072**	-0.030	-0.040	-0.116***	-0.034*
	(0.017)	(0.017)	(0.029)	(0.029)	(0.028)	(0.038)	(0.019)
ITT 2013	-0.017	-0.017	-0.022	-0.016	-0.027	-0.048	-0.010
	(0.019)	(0.019)	(0.031)	(0.035)	(0.028)	(0.047)	(0.021)
N	3117	3096	1034	1032	1030	678	2418
Adj. R-sq	0.007	0.009	0.016	0.008	0.006	0.013	0.008
**Panel B: CHE (>=40% of capacity to pay)**							
ITT 2011	-0.008	-0.009	-0.019	-0.003	-0.012	-0.038	-0.003
	(0.015)	(0.015)	(0.030)	(0.021)	(0.014)	(0.045)	(0.017)
ITT 2013	-0.020	-0.022	-0.066	-0.009	-0.010	-0.031	-0.020
	(0.020)	(0.020)	(0.041)	(0.031)	(0.019)	(0.054)	(0.021)
N	3117	3096	1034	1032	1030	678	2418
Adj. R-sq	0.007	0.025	0.030	0.011	0.006	0.029	0.024

Standard errors clustered at EA level (in parentheses)

* p<0.10; ** p<0.05; *** p<0.01

Based on the balanced panel of 1,039 households.

Observations are inverse-probability weighted to correct for attrition on observables. Covariates included in the ITT regressions are gender and marital status of the household head, and household size.

Table 3: Impact of health insurance on CHE for full sample and different thresholds of CHE, [(Intention to Treat 2011 and 2013)]

	Percentage of household consumption					Percentage of CTP			
	(1)<br>5%	(2)<br>10%	(3)<br>15%	(4)<br>20%	(5)<br>25%	(6)<br>10%	(7)<br>20%	(8)<br>30%	(9)<br>40%
ITT 2011	-0.107***	-0.043**	-0.024**	-0.014*	-0.007	-0.075***	-0.021	-0.018	-0.007
	(0.025)	(0.017)	(0.011)	(0.008)	(0.007)	(0.027)	(0.019)	(0.014)	(0.011)
ITT 2013	-0.024	-0.009	-0.005	-0.001	0.002	-0.032	-0.011	-0.018	-0.011
	(0.022)	(0.015)	(0.012)	(0.008)	(0.007)	(0.029)	(0.021)	(0.016)	(0.013)
N	3096	3096	3096	3096	3096	3096	3096	3096	3096
Adj R-squared	0.008	0.006	0.003	-0.000	-0.001	0.055	0.027	0.022	0.020

Standard errors clustered at EA level (in parentheses)
* p<0.10; ** p<0.05; *** p<0.01
Based on the balanced panel of 1,039 households.
Covariates included in the ITT regressions are gender and marital status of the household head, and household size.

Table 4: Household Insurance Status Logit regressions

	Insurance status in 2011	Insurance status in 2013	Insurance status in 2011	Insurance status in 2013
Age of HH head	0.003	0.006	0.003	0.005
	(0.007)	(0.006)	(0.007)	(0.006)
Household head is Female	0.181	0.665**	0.198	0.672***
	(0.260)	(0.262)	(0.259)	(0.260)
Dummy: household head is married	0.433	0.737***	0.437	0.745***
	(0.314)	(0.268)	(0.316)	(0.273)
Urban	0.282	-0.319	0.293	-0.315
	(0.425)	(0.484)	(0.424)	(0.485)
HH head: At least primary school education	0.123	0.070	0.130	0.073
	(0.272)	(0.180)	(0.275)	(0.182)
Distance (km) to nearest HCHP clinic	-0.234***	-0.212**	-0.231***	-0.211**
	(0.087)	(0.101)	(0.087)	(0.101)
Household size	-0.047	0.053	-0.046	0.055
	(0.049)	(0.042)	(0.048)	(0.042)
Log (HH Annual household consumption corrected for inflation)	0.258	0.081	0.267	0.095
	(0.173)	(0.222)	(0.175)	(0.224)
Good quality toilet	1.932*	0.529	1.942*	0.534
	(1.159)	(0.717)	(1.163)	(0.718)
Good quality drinking water	-0.124	-0.031	-0.127	-0.030
	(0.230)	(0.263)	(0.231)	(0.266)
Log (HH annual health expenditures excl. premium)	0.018	0.081**	0.013	0.075**
	(0.036)	(0.034)	(0.036)	(0.034)
Dummy: All HH members can do daily activities without difficulty	-0.065	0.087	-0.067	0.085
	(0.174)	(0.190)	(0.172)	(0.191)
Dummy: at least 1 HH member has chronic disease in past 12 months	-0.075	-0.103	-0.083	-0.116
	(0.188)	(0.179)	(0.187)	(0.181)
Dummy: at least 1 HH member had an acute illness/injury in past 12 months	-0.115	-0.232	-0.108	-0.226
	(0.173)	(0.202)	(0.173)	(0.204)
Dummy: Catastrophic Heath Expenditure (threshold of >10% of household consumption) at baseline	-0.587	-0.332		
	(0.596)	(0.592)		
Dummy: Catastrophic Heath Expenditure (threshold of >40% of Capacity to pay) at baseline			-0.118	0.380
			(0.648)	(0.772)
Constant	-3.415	-1.991	-3.531	-2.153
	(2.263)	(2.900)	(2.286)	(2.923)
Number of Observations	672	672	672	672

* *p*<0.1; ** *p*<0.05; *** *p*<0.01; Standard errors in parenthesis, clustered at EA level.

Table 5: Impact of health insurance on CHE for full sample, by wealth terciles and by chronic illness status with attrition weights, [(Instrumental Variable Approach 2011 and 2013)]

	(1) **Full sample -no controls**	(2) **Full sample-controls**	(3) **Poor**	(4) **Middle**	(5) **Rich**	(6) **Chronic**	(7) **Non-Chronic**
**Panel A: CHE (> 10% of household consumption)**							
Household Insured 2011	-0.112***	-0.119***	-0.192*	-0.028	-0.089**	-0.196**	-0.106***
	(0.034)	(0.036)	(0.100)	(0.053)	(0.035)	(0.097)	(0.038)
Controls	*No*	*Yes*	*Yes*	*Yes*	*Yes*	*Yes*	*Yes*
N	1036	1036	347	346	344	226	810
Household Insured 2013	-0.035	-0.035	-0.018	-0.058	-0.049*	-0.000	-0.039
	(0.034)	(0.033)	(0.069)	(0.052)	(0.029)	(0.079)	(0.036)
Controls	*No*	*Yes*	*Yes*	*Yes*	*Yes*	*Yes*	*Yes*
N	1039	1039	347	346	346	227	812
**Panel B: CHE (>=40% of capacity to pay)**							
Household Insured 2011	0.010	0.008	0.034	-0.015	-0.041*	0.069	-0.001
	(0.030)	(0.032)	(0.096)	(0.022)	(0.022)	(0.101)	(0.033)
Controls	*No*	*Yes*	*Yes*	*Yes*	*Yes*	*Yes*	*Yes*
N	1036	1036	347	345	344	226	810
Household Insured 2013	-0.016	-0.013	-0.060	-0.028	-0.026	0.062	-0.025
	(0.041)	(0.040)	(0.097)	(0.053)	(0.021)	(0.087)	(0.044)
Controls	*No*	*Yes*	*Yes*	*Yes*	*Yes*	*Yes*	*Yes*
N	1039	1039	347	346	346	227	812

Standard errors clustered at EA level (in parentheses)
* p<0.10; ** p<0.05; *** p<0.01
Based on the balanced panel of 1,039 households.
Covariates included in regressions are gender and marital status of household head, household size.

Table 6: Impact of health insurance on CHE for full sample, by wealth terciles and by Illness status [(Intention to treat estimates 2011 and 2013)] using probit regressions

	(1) **Full sample -no controls**	(2) **Full sample-controls**	(3) **poor**	(4) **middle**	(5) **rich**	(6) **chronic**	(7) **Non-chronic**
**Panel A: CHE (> 10% of household consumption)**							
ITT 2011	-0.601**	-0.591**	-0.864**	-0.358	-0.515	-1.525***	-0.299
	(0.271)	(0.268)	(0.388)	(0.413)	(0.371)	(0.550)	(0.278)
ITT 2013	-0.130	-0.121	0.000	-0.133	-0.242	-0.465	0.011
	(0.257)	(0.258)	(.)	(0.427)	(0.448)	(0.546)	(0.288)
N	3117	3096	954	1032	1030	678	2418
Adj. R-sq	0.007	0.009	0.016	0.008	0.006	0.013	0.008
**Panel B: CHE (>=40% of capacity to pay)**							
ITT 2011	-0.222	-0.199	0.571	-0.296	0.000	-0.290	0.223
	(0.308)	(0.334)	(0.443)	(0.706)	(.)	(0.464)	(0.371)
ITT 2013	-0.263	-0.238	0.000	-0.325	0.000	0.000	0.018
	(0.269)	(0.300)	(.)	(0.542)	(.)	(.)	(0.313)
N	3117	3096	954	1032	672	592	2418
Adj. R-sq	0.007	0.025	0.030	0.011	0.006	0.029	0.024

Standard errors clustered at EA level (in parentheses)
* p<0.10; ** p<0.05; *** p<0.01
Based on the balanced panel of 1,039 households.
Covariates included in the ITT regressions are gender and marital status of the household head, and household size.
